# Maternal Protein Restriction Modulates Angiogenesis and AQP9 Expression Leading to a Delay in Postnatal Epididymal Development in Rat

**DOI:** 10.3390/cells8091094

**Published:** 2019-09-17

**Authors:** Talita de Mello Santos, Marilia Martins Cavariani, Dhrielly Natália Pereira, Bruno César Schimming, Luiz Gustavo de Almeida Chuffa, Raquel Fantin Domeniconi

**Affiliations:** Department of Anatomy, Institute of Biosciences, São Paulo State University-UNESP, Botucatu 18618-970, SP, Brazil; marycavariani@hotmail.com (M.M.C.); dhrielly.p@gmail.com (D.N.P.); bruno.schimming@unesp.br (B.C.S.); luiz-gustavo.chuffa@unesp.br (L.G.d.A.C.); raquel.domeniconi@unesp.br (R.F.D.)

**Keywords:** fetal programing, aqp1, aqp9, vegf, protein restriction, epididymal development

## Abstract

The maternal nutritional status is essential to the health and well-being of the fetus. Maternal protein restriction during the perinatal stage causes sperm alterations in the offspring that are associated with epididymal dysfunctions. Vascular endothelial growth factor (VEGF) and its receptor, VEGFr-2, as well as aquaporins (AQPs) are important regulators of angiogenesis and the epididymal microenvironment and are associated with male fertility. We investigated the effects of maternal protein restriction on epididymal angiogenesis and AQP expression in the early stages of postnatal epididymal development. Pregnant rats were divided into two experimental groups that received either a normoprotein (17% protein) or low-protein diet (6% protein) during gestation and lactation. At postnatal day (PND)7 and PND14, male offspring were euthanized, the epididymides were subjected to morphometric and microvascular density analyses and to VEGF-A, VEGF-r2, AQP1 and AQP9 expression analyses. The maternal low-protein diet decreased AQP9 and VEGFr-2 expression, decreased epididymal microvascularity and altered the morphometric features of the epididymal epithelium; no changes in AQP1 expression were observed at the beginning of postnatal epididymal development. Maternal protein restriction alters microvascularization and affects molecules involved in the epidydimal microenvironment, resulting in morphometric alterations related to a delay in the beginning of epididymis postnatal development.

## 1. Introduction

Studies have revealed potential factors that increase the risks of diseases during the developmental period unpublished data [1,2,3]. These studies support the developmental origins hypothesis, which is based on the interaction between early developmental plasticity and environmental factors, and studies have been conducted to determine the mechanisms by which these factors may affect health and disease later in life [4,5]. The genome is evolutionarily and chemically very stable, so environmental factors generally do not cause genomic changes in DNA sequences. These factors may cause hereditary changes through manipulation independent of DNA sequence, thereby changing the epigenome. These alterations may be due to DNA methylation, histone acetylation, hormonal imprinting and/or growth factors and transcription alterations [6,7]. Poor-quality nutrition during the pre- and postnatal periods is an extrinsic maternal factor and has been shown to be a key factor in the development of several diseases [8,9,10]. In recent years, male infertility has been shown to be associated with congenital defects caused by embryonic and fetal development [11]. Epidemiological and experimental evidence has shown an association between low birth weight and male subfertility in humans [12].

Boeri et al. (2016) [13] observed a positive correlation between infertile adults born with a low birth weight. These adult males had alterations associated mainly with epididymal functions, such as reduced sperm motility, including asthenozoospermia (reduction or absence of sperm motility) and higher rates of teratozoospermia (alterations in sperm morphology that may lead to infertility). Of the fetal programming models for maternal nutrition, that of protein restriction in a diet offered to pregnant rats is one of the most frequently used, leading to increased risks of certain diseases or disorders later in life as cardiovascular and metabolic diseases [14,15]. 

The protein restriction diet in pregnant rats has already been shown to be able to alter maternal metabolism, increasing maternal serum corticosterone, estradiol and testosterone concentrations. In addition, other studies observed alterations in seminiferous tubule organization and in epididymal functions, similar to those found in men with low birth weight. These alterations include reductions in sperm motility, sperm concentration and viability, in addition to morphological changes [16,17].

In male embryos, the proximal region of the mesonephric tubules originates the mesonephric ducts or Wolffian ducts (WD) that eventually become the epididymis. During embryonic and postnatal development, these ducts elongate and acquire a coiled aspect under the influence of testosterone and growth factors; these factors regulate gene expression and epithelial-mesenchymal interactions [18,19,20]. During postnatal development, the rat epididymis grows and expands, and the epithelial and mesenchymal cells differentiate into several cell types. This cell differentiation period consists of an undifferentiated stage between the 1st and 15th PND, followed by a differentiation phase, when the first spermatozoa are observed after the first testicular fluid wave (between the 15th and 45th PND). In brief, the arrival of these growth factors during the first stage of postnatal development occurs through the blood vessels [21].

The epididymis is usually divided into four regions, namely, the initial segment (IS), caput, corpus and cauda, and each region contributes in an essential way to the process of sperm maturation [22]. However, the most proximal region of the epididymis, the IS, may undergo proper differentiation to ensure its function in sperm maturation, being the region of the epididymis that is critical for male fertility; the incomplete development of this region results in infertility [23].

Because of the relationship between capillary flow volume and the local cell metabolic rate, blood flow into the IS is higher than in any other region of the epididymis. Therefore, this segment is metabolically very active and is dependent on factors secreted directly by the testes and on circulating androgens [24]. Vascular endothelial growth factor (VEGF) and VEGF receptor 2 (VEGFr-2) are the main regulators in angiogenesis promotion and are essential for male fertility. Studies have shown an association between VEGF expression and male infertility associated with blood vessel formation. Recent evidence has indicated that these factors affect the epididymal microenvironment and sperm maturation [25].

Another important group of proteins related to the epididymal microenvironment is composed of the aquaporins (AQPs). AQPs are transmembrane proteins involved in fluid reabsorption/secretion dynamics, which are essential for the regulation of water homeostasis. Nine mRNAs that code for AQPs (Aqp1–3, 5–9 and 11) are present in the epididymis [26]. Among these, stand out AQP1 and AQP9 are expressed in endothelial cells and muscle cells and in principal cells, respectively. While AQP1 is present in the endothelium of vascular channels and is important for maintaining fluid equilibrium is the tissue, AQP9 is the main AQP of the epididymal epithelium, where it contributes to apical membrane permeability to water and neutral solutes. The AQP9 promoter contains a putative steroid receptor binding site and is affected by the estrogen/androgen balance. Conversely, AQP1 does not seem to be directly affected by sex hormones [27,28].

A recently published study by our research group showed that some impairments that appeared at puberty in animals whose mothers received a low-protein diet during gestation and lactation, such as changes in AQP1, AQP9, VEGFa and VEGFR-2 expression and in the microvasculature density (MVD) [29], were alleviated when the animals reached adulthood. Interestingly, sperm alterations, which are associated with epididymal functions, are still observed in these adult animals [16].

To characterize the involvement of functional alterations in the adult epididymis, it is necessary to investigate the MVD in all epididymal regions and the expression of AQPs, VEGFa and VGFR-2 during early postnatal epididymal development. The results may contribute new data to the field of reproductive biology and clarify possible gaps in epididymis-related low fertility related after maternal protein restriction.

## 2. Materials and Methods

### 2.1. Experimental Design

Adult female (60 days of age, *n* = 40) and male (90 days of age, *n* = 20) Wistar rats were obtained from the Central Biotherium of São Paulo State University (UNESP) and housed in polypropylene cages (43 cm × 30 cm × 15 cm) with laboratory grade pine shavings as bedding. The rats were maintained under controlled temperature (22 ± 2 °C), relative humidity (55 ± 10%) and lighting conditions (12:12-h photoperiod). Balanced rat chow (NUVILAB^®^ CR1–Nuvital-PR) and filtered tap water were provided ad libitum. The experimental protocol was designed following Brazilian laws and was approved by the Ethical Committee on Animal Use from the Institute of Biosciences of Botucatu (CEUA: protocol n° 797).

Two female rats were mated with one male during the dark period of the light/dark cycle, and the following day, vaginal smears were performed. The day of detection of the sperm in the vaginal smear was considered gestational day 0 (GD 0). Pregnant females were randomly allocated into 2 experimental groups: The normoprotein group (NP, *n* = 19) and the low-protein group (LP, *n* = 19). The NP pregnant females were fed a normoprotein diet (17% protein) ad libitum, and the LP pregnant females were fed a low-protein diet (6% protein) ad libitum. Both groups received the diet over the entire gestational and lactation periods (Table 1).

The pregnant females were housed in individual cages and weighed every week to monitor weight gain. During the entire gestation period until PND14, the total food consumed was monitored weekly, and the values obtained were divided by seven to estimate the daily consumption of each animal in both groups.

**Figure 1:**
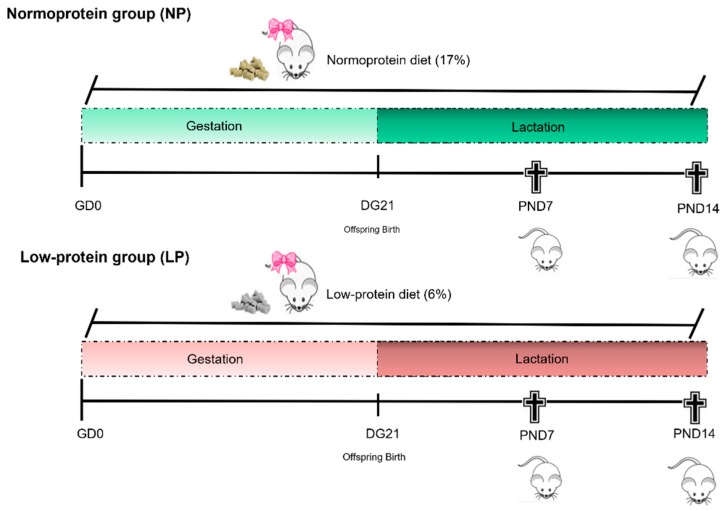
Illustration of the experimental design.

After birth, the litter size was standardized to eight pups per mother in both experimental groups to ensure the equality of the weaning to all offspring. The pups remained with the mothers in individual boxes until the moment of euthanasia at PND7 (NP = 17 pups/group; LP = 19 pups/group) or PND14 (NP = 19 pups/group; LP = 26 pups/group) and had their body weights and lengths measured before euthanasia. After the pups were anesthetized with ketamine and xylazine, they were killed by decapitation, and the epididymides were collected. After being weighed, the left epididymides were immediately packed in cryotubes, frozen in liquid nitrogen and stored at −80 °C. The right epididymides were collected, dissected and fixed in 10% buffered formalin (0.1 M phosphate buffer pH 7.3) for 24 h, washed in running water for 24 h and stored in 70% alcohol (Figure 1).

### 2.2. Western Blot 

Total epididymides were used to perform western blotting. Due to the small size and weight of the epididymis, pools were used with 4–5 animals/pool for a total of 5 samples/group for animals at P7, and 2–3 animals/pool for a total of 5 samples/group for animals at P14. The samples were homogenized at 4 °C in RIPA buffer (BioRad^®^, Hercules, CA, USA) with protease inhibitor (Sigma-Aldrich^®^, St. Louis, MO, USA) with a Tureaux-type homogenizer for 3 5-second cycles. The homogenate was centrifuged at 15,000 rpm for 20 min at 4 °C, and the supernatant was collected. The protein quantification was performed as described by Bradford [30] in ELISA plates with 96 wells and read in an ELISA reader (595 nm). Aliquots (70 µg protein) were treated with buffer solution (Laemli Sample Buffer-BioRad^®^, Hercules, CA, USA) and β-mercaptoethanol at 95 °C for 5 min. Then, the proteins were separated by vertical SDS-PAGE (Mini-Protean, BioRad^®^, Hercules, CA, USA). After electrophoresis, proteins were transferred to a nitrocellulose membrane in a wet transfer system. Nonspecific protein binding was blocked with 3% skim milk in TBST buffer for 1 h at room temperature. Then, the membranes were incubated overnight with the following primary antibodies diluted in TBST: AQP1 (concentration 1:800; AB2219 EMD-Millipore Corp.^®^, Billerica, MA, USA); AQP9 (concentration 1:500, APQ91-A Alpha Diagnostic, San Antonio, TX, USA); VEGFa (concentration 1:1000, SC-152 Santa Cruz, Dallas, TX, USA); VEGFr-2 (concentration 1:1000, SC-6251 Santa Cruz, Dallas, TX, USA); and β-actin (concentration 1:800; ab-8227 Abcam Inc.^®^, Cambridge, MA, USA). Subsequently, the membranes were washed for three times for 10 min each in TBST and incubated for 2 h with the following specific HRP secondary antibodies diluted in TBST: IgG goat-anti rabbit for AQP1, AQP9 (concentration 1:5000, Sigma-Aldrich Co.^®^, St. Louis, MO, USA) and VEGFa (concentration 1:2000, Sigma-Aldrich Co.^®^, St. Louis, MO, USA); IgG goat-anti mouse for VEGFr-2 (concentration 1:5000, Sigma-Aldrich Co.^®^, St. Louis, MO, USA); and IgG rabbit-anti goat for β-actin (concentration 1:6000, Sigma-Aldrich Co.^®^, St. Louis, MO, USA). The membranes were then washed three times for 10 min each with TBST. Immunoreactive components were visualized with a chemiluminescence kit (Amersham^TM^ ELC Select^TM^ Western Blotting Detection Reagent, GE Healthcare^®^, Chicago, IL, USA), and the optical density of each band was measured by ImageJ^®^ Windows^®^ software, normalized to β-actin.

### 2.3. Morphometrical Analysis

The epididymides fixed in 10% buffered formalin were cleared in xylene and then embedded in Paraplast. Serial 4-µm sections were obtained with an automatic microtome and added to silanized slides. Histological sections of tissue from animals in different litters (*n* = 4) were stained with hematoxylin and eosin (HE) and scanned using a 3D Histech Pannoramic MIDI. Thereafter, tissues were analyzed and photographed, and the luminal and tubular diameter and the epithelium length from the IS, caput, corpus and cauda were measured using the Panoramic Viewer program.

### 2.4. Immunohistochemistry at PND 14

Epididymal sections from the LP and NP animals (sections of animals from different litters; *n* = 4 animals/group) were subjected to antigen retrieval in a humid environment (electric pot) at 100 °C in Tris/0.1 M EDTA pH 9.0 for 30 min. After being washed in distilled water, the sections were subjected to the blocking of endogenous peroxidase (3% hydrogen peroxide in methanol) for 15 min. To block nonspecific binding, the slides were incubated with 3% skim milk in PBS for 1 h. Then, the sections were incubated overnight (at 4 °C) with primary antibodies to AQP1 (concentration 1:200; AB2219 EMD-Millipore Corp.^®^, Billerica, MA, USA) or AQP9 (concentration 1:200, APQ91-A Alpha Diagnostic, San Antonio, TX, USA), which were diluted in 1% BSA. After incubation with the primary antibodies, the sections were washed in PBS and then incubated with anti-rabbit secondary HRP antibody (ab97051; concentration 1:200, Abcam Inc.^®^, Cambridge, MA, USA) for 2 h at room temperature. The reaction was visualized with DAB chromogen (3,3′-diaminobenzidine tetrahydrochloride; Sigma-Aldrich Co.^®^, St. Louis, MO, USA) and counterstained with hematoxylin for 1 min. The slides were scanned using a 3D Histech Pannoramic MIDI (3DHistech, Budapest, Pest, Hungary) and analyzed and photographed using the Pannoramic Viewer program.

### 2.5. Microvascular Density (MVD) Determination at PND14

The MDV was determined per the procedures described by Cavariani et al., 2019 [29]. Epididymal sections from LP and NP animals (*n* = 4 animals/group) that had been immunostained with anti-AQP1 were evaluated by stereological analysis to estimate the number of microvessels in the IS, caput, corpus and cauda epididymis. Data are presented as the percentage of microvessels per total stromal area analyzed.

### 2.6. Statistical Analysis

*T*-tests for parametric data and the Mann–Whitney test for nonparametric data were performed. The data were compared among experimental groups using GraphPad Prism^®^ software (version 5.00). Differences were considered significant when *p* ≤ 0.05. Data are presented as the mean ± standard error of the mean (SEM).

## 3. Results

### 3.1. A Maternal Low-Protein Diet Alters the Protein Levels of VEGFr-2 and VEGFa in the Epididymis of the Offspring in the First Postnatal Week

**Figure 2:**
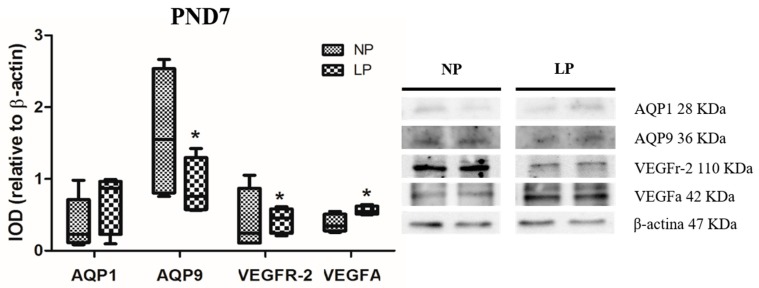
Expression of AQP1, AQP9, VEGFr-2 and VEGFa in the epididymis of NP and LP animals at PND7. Data are expressed as the mean ± S.E.M. * *p* < 0.05. Mann–Whitney test.

**Figure 3:**
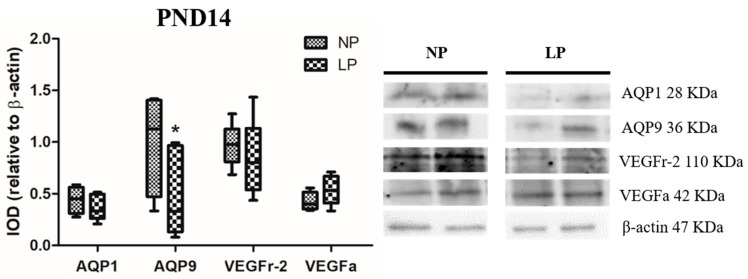
Expression of AQP1, AQP9, VEGFr-2 and VEGFa in the epididymis of NP and LP animals at PND14. Data are expressed as the mean ± S.E.M. * *p* < 0.05. Mann–Whitney test.

The levels of VEGFa were increased (fold-change 1.47) and those of VEGFr-2 were decreased (fold-change 0.95) in LP animals compared with NP animals at PND7 (Figure 2). However, the levels of VEGFa and VEGFr-2 did not change significantly at PND14 (fold-change increase of 1.03 and decrease of 0.85, respectively) (Figure 3).

### 3.2. A Maternal Low-Protein Diet Alters the Protein Levels of AQP9 but Not of AQP1 in the Epididymis of the Offspring at PND7 and PND14

The animals whose mothers were subjected to protein restriction during gestation and lactation presented a reduction in the protein levels of AQP9 at PND7 (fold-change 0.54) and PND14 (fold-change 0.33), but no change was observed for AQP1 in the epididymis at any age studied (fold-change PND7 1.73 and PND14 1.07) (Figure 2 and Figure 3).

### 3.3. A Maternal Low-Protein Diet Promotes Changes in the Immunostaining Pattern of AQP9 but Not of AQP1 in the Offspring at PND14

**Figure 4:**
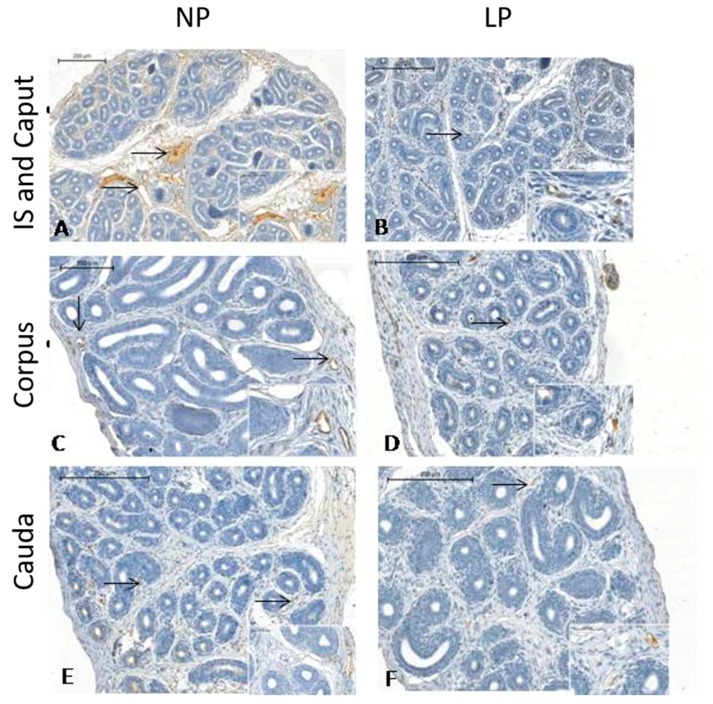
Epididymis sections of the initial segment (IS), caput regions, corpus and cauda from the NP and LP animals at PND14 subjected to AQP1 immunostaining. NP = normoprotein animals; LP = low-protein animals. (**A**) (IS and Caput from NP group); (**B**) (IS and Caput from LP group); (**C**) (Corpus from NP group); (**D**) (Corpus from LP group); (**E**) (Cauda from NP group); (**F**) (Cauda from LP group). Arrow = Positive staining for AQP1 in the vascular endothelium.

**Figure 5:**
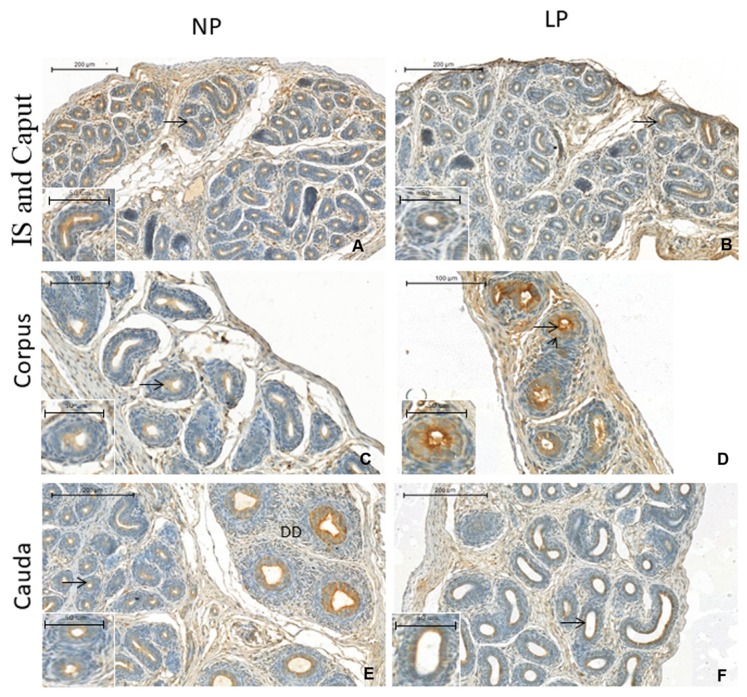
Epididymis sections of the initial segment (IS), caput regions, corpus and cauda from the NP and LP animals at PND14 subjected to AQP9 immunostaining. NP = normoprotein animals; LP = low-protein animals. (**A**) (IS and Caput from NP group); (**B**) (IS and Caput from LP group); (**C**) (Corpus from NP group); (**D**) (Corpus from LP group); (**E**) (Cauda from NP group); (**F**) (Cauda from LP group). Arrow = Positive staining for AQP9.

This study is the first to demonstrate AQP1 and AQP9 positive staining in the epididymis of rats aged only 14 days (Figure 4 and Figure 5, respectively). The youngest age of rats examined to date was PND 21, which was recently reported by our research group [29]. Interestingly, the immunolocalization pattern of AQP1 was the same as that described by Cavariani et al., 2019 [29]. AQP1 staining appeared in the endothelial cells of vascular channels throughout the epididymis in both the NP and LP groups, but staining was not observed in the peritubular cells that surrounded the duct, probably because the mesenchymal cells were not differentiated into muscle cells yet. No differences were observed between the NP and LP groups. However, we noticed a lower number of vessels in the LP group than in the NP group in all regions, but mainly in the IS region (Figure 4B); this was the most vascularized epididymis region compared to the same region in the NP animals (Figure 4A). We could also verify that the blood vessels of the diet-restricted animals had a smaller diameter, showing that the development of these vessels was delayed (Figure 4). AQP9 immunolocalization showed discontinuous immunostaining in different regions of the epididymis, with AQP9 being positively localized to the apical region of the epithelial cells (Figure 5D). In the region of the IS (Figure 5A,B), in both the NP and LP groups, there was stronger staining than in the adjacent (caput) region. However, this staining was more intense in the IS of the NP group than in the IS of the LP group. In the epididymal corpus of the LP animals (Figure 5D), the intensity of AQP9 staining was stronger than that in the NP group (Figure 5C). In the caput region (Figure 5E,F), there was discontinuous immunostaining of AQP9 in the tubular epithelium of both groups, with staining being more prevalent in the transition region of the vas deferens.

### 3.4. A Maternal Low-Protein Diet Decreases the Microvascular Density (MVD) of the Offspring at PND14

**Figure 6:**
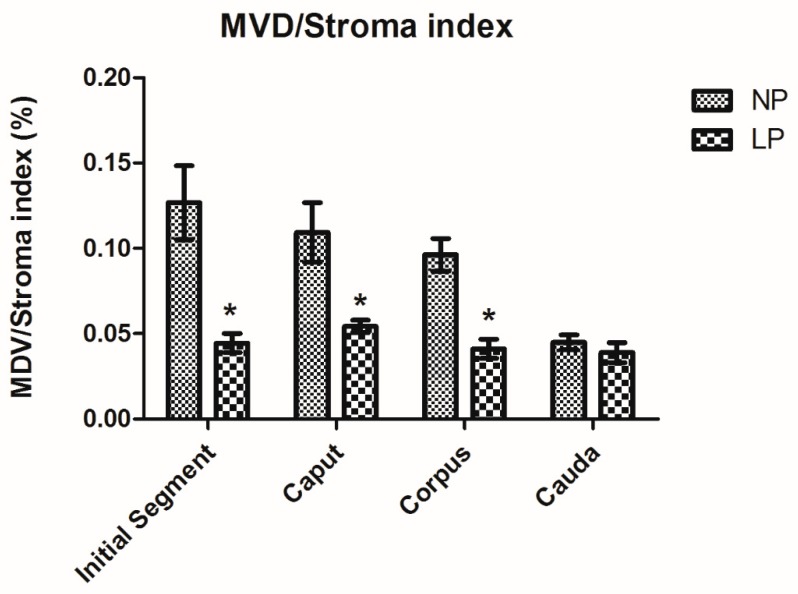
The microvascular densities in the epididymis of the NP and LP animals at PND14. Data are expressed as the mean ± S.E.M. * *p* < 0.05. Mann–Whitney test.

The MVD/stroma index was used to analyze the epididymis microvasculature. These values showed a decrease in the blood supply of the IS, caput and corpus, but not in the cauda at PND14 in the LP animals compared to the NP group (Figure 6).

### 3.5. A Maternal Low-Protein Diet Changes the Epididymal Morphometry of the Offspring but Not the Morphology

The 7-day-old LP epididymis showed a decrease in tubular diameter in the IS, caput and corpus, but not in the cauda; the luminal diameter was decreased only in the IS (Table 2). The epithelial height was decreased in the IS and corpus, and at PND14, the tubular diameter was decreased in the epididymis corpus of the LP animals but not in that of the NP animals (Table 3). A normal epididymal structure was observed in both groups at PND7 and PND14. The epididymal ducts were lined by a columnar epithelium resting on a basal lamina. A few halo cells were found among the columnar cells. The epididymal ducts were surrounded by a thin layer of mesenchymal cells that had not differentiated into smooth muscle (Supplementary Materials).

## 4. Discussion

**Figure 7:**
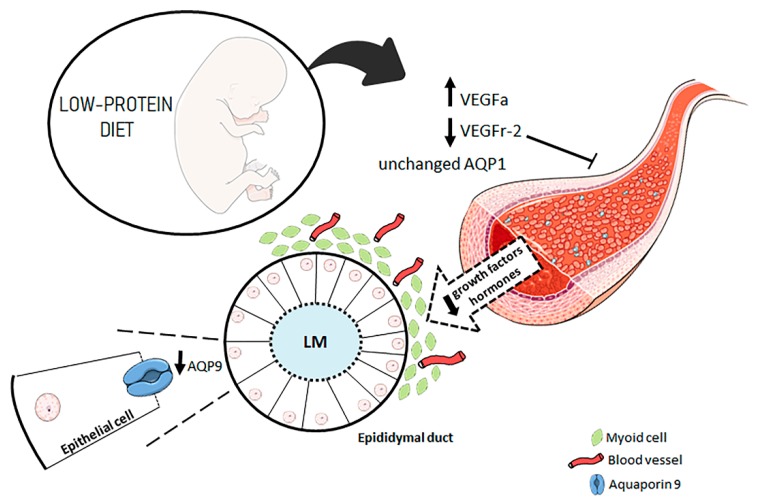
Representative image that illustrates the impact of maternal protein restriction in the early stage of postnatal epididymal development in rat. Maternal protein restriction decreases microvascular density by decreasing the VEGFr-2 expression in the endothelial cells. With the decrease in the number of vessels, the arrival of growth factors and hormones, including androgens, also decreases. Blood vessels are present around the mesenchymal cells that surround the epithelium, and before the first wave of testicular fluid, the arrival of hormones occurs exclusively through blood supply. When differentiated, the principal cells begin to express AQP9 in the apical region. Because AQP9 is downregulated in these cells, the luminal microenvironment may not be adequately regulated. AQP9 = aquaporin 9; AQP1 = aquaporin 1; VEGFa = vascular endothelial growth factor; VEGFr-2 = vascular endothelial growth factor receptor type 2, LM: luminal microenvironment.

In the present study, we showed that a maternal low-protein diet during gestation and lactation was able to decrease AQP9 and VEGFr-2 expression while enhancing VEGFA expression, changing the patterns of epididymal microvascularity and altering the features of the epididymal epithelium (Figure 7).

According to the developmental origins of health and diseases (DOHaD) hypothesis, the high plasticity of the fetal organism is conditioned by an adaptive response that can modify the individual phenotype. The interactions between intrinsic factors, such as genetics and epigenetics, and extrinsic maternal factors, such as maternal nutrition, can influence these developmental processes and lead to permanent changes in adulthood [31,32,33].

During postnatal development, the epididymal duct elongates and coils, and the connective tissue septa start to divide the epididymis into different segments [21]. At the same time, the epididymal vasculature grows to follow the development of the epididymal duct and ensure vascularization of the expanding organ [34].

VEGFa is an important growth factor associated with the reproductive system and acts through VEGFr-2 to promote angiogenesis; VEGFa and VEGFr-2 are mostly expressed in the initial segment [24,35]. Both VEGFa and VEGFr-2 are specific endothelial cell factors that participate in sperm maturation through their important roles in maintaining the epididymal microenvironment and promoting cell proliferation, migration and tube formation in normal epididymal tissues [25,36].

Although the VEGFr-2 and VEGFa levels were unchanged in LP animals at PND14, we observed a decrease in VEGFr-2 expression in the diet-restricted animals at PND7. This result could be associated with a decrease in microvascular density (MVD), especially in the initial segment that was observed in the same group at PDN14. Thus, a maternal low-protein diet was able to alter the epididymal vascular pattern during an important period of the postnatal development of this organ.

Maternal protein malnutrition has already been shown to be associated with an impairment in angiogenesis with decreased VEGFr-2 expression in the prostate. Previous work has shown that maternal protein malnutrition leads to a decrease in VEGFr-2 expression in the prostate, which compromises the angiogenesis process. This result was associated with a delay in prostate development at PND10 and PND21 in rat offspring from mothers receiving a protein-restricted diet during gestation and lactation [37].

During the early postnatal period, an adequate blood supply is crucial to promote the transport of hormones, metabolites and nutrients required by the epididymis to maintain its normal development [38]. A reduction in MVD can lead to ineffective diffusion of hormones, growth factors and oxygen, resulting in altered production of stimulatory molecules related to cellular differentiation and morphogenesis, leading to permanent effects in adulthood [37]. Recently, we showed that maternal protein restriction resulted in a low MVD in all epididymal regions at the prepubertal stage. However, this decreased pattern of vascularization did not remain in adulthood, and MVD was restored in LP animals, maintaining their MVD values to the levels of those found in the NP group at PND120 [29].

VEGFa is required for the formation of blood vessels during fetal development, while in adults, VEGFa is necessary to maintain the regular function of blood vessels. The increase in VEGFa expression during epididymal development is important to promote vascular permeability and provide a blood supply due to the absence of neovascularization in this organ during adulthood [39]. We hypothesized that the recovery of MVD in adulthood may be associated with the increase in VEGFa expression observed in LP animals at the beginning of epididymal development (PND7), as this growth factor acts as a paracrine effector that regulates the division of blood vessel cells in an attempt to stimulate neovascularization in the epididymis of restrict animals.

In addition to VEGF, AQPs are also involved in angiogenesis. Mainly through water homeostasis, AQPs are essential regulators of epididymal function, contributing to membrane fluidity and structural integrity [28,40,41]. Recent evidence indicates that AQPs are involved in cell migration, proliferation, carcinogenesis and physiologic inflammatory processes [42]. Several studies have associated different diseases with changes in AQP mRNA and protein expression. It has been suggested that AQP1 is involved in lung cancer differentiation [43] and in the pathophysiology of inflammatory bowel disease [44]. AQP9 is associated with ovarian cancer [45] and hepatocellular carcinoma [46], in addition to inflammatory diseases such as rheumatoid arthritis [47] and, more recently, endometriosis [48].

In the epididymis, AQP9 is responsible for removing fluid from the epididymal lumen, while AQP1 removes fluid from the epididymis to the blood vessels, both of which play a crucial role in the secretion/reabsorption dynamics during sperm transport and maturation [49].

AQP1 is a water-selective channel that is permeable to water but not to small organic and inorganic ions/molecules [50]. It is an important structural protein present in the endothelial cells of blood vessels and, in adult rats, is also found in the smooth muscle cells that surround the epididymal duct [27,51,52]. There are no previous studies showing immunostaining for AQP1 in the epididymis of young rats due to the absence of this protein in the early developmental stages of this organ. Thus, we were not able to observe AQP1 labeling in the 7-day-old animals. However, it is important to reinforce that this is the first study to show AQP1 immunostaining at PND14.

At PND14, AQP1 immunolocalization was observed in the endothelial cells of vascular channels in both the NP and LP animals, as described in 21-day-old rats [29]. The staining was not found in the smooth muscle cells, as was observed in the adult epididymis, probably because the mesenchymal cells are not differentiated into muscle cells due to a period of no differentiation [26,29]. There was no difference in the immunostaining of this protein between the experimental groups in any of the epididymal regions. However, we observed a higher number of AQP1-positive vessels in the NP group, mainly in the IS, than in the LP group. This was confirmed by the MVD analyses. Moreover, we observed that the diameter of the vessels was smaller in this diet-restricted group, showing a delay in vessel development. However, more in-depth studies that specifically evaluate angiogenesis are necessary.

Maternal protein restriction causes a delay in prostate morphogenesis in male rats at PND10 and PND21 that is associated with a reduced angiogenic process through downregulation of the AQP1 and VEGF signaling pathways [37]. However, our data showed that this low-protein fetal programming did not change AQP1 levels at the beginning of epididymis postnatal development. It is likely that the maintenance of the AQP1 levels in the LP group, similar to the NP group, is a possible mechanism to preserve vascular epididymal permeability despite the decrease in the MVD.

AQP9 is considered an aquaglyceroporin due to its permeability characteristics that allow the passage of a wide range of structurally unrelated molecules, including urea, glycerol, mannitol, sorbitol and water [53]. In the epididymis, this protein is the main AQP that contributes to organ permeability and is located in the stereocilia of epididymal principal cells [26,28,54].

Studies have shown that AQP9 mRNA levels can be found between birth and the 3rd week during postnatal epididymal development in rats. However, only after the first 4th postnatal week did AQP9 protein expression begin to increase significantly, becoming abundant in adulthood [27,55].

As mentioned for AQP1, this is the first study to examine the immunolocalization of AQP9 at PND14. AQP9 staining was observed in epididymal epithelial cells that began differentiation into principal cells. Apparently, this staining was weaker at PND14 than that observed in adult rats [28,29]. In addition, comparing both experimental groups, we observed weak staining in principal cells in LP animals, mainly in the proximal epididymal region.

Despite the absence of AQP9 immunolabeling at PND7, we were able to observe the expression of this protein at both ages due to its sensitivity. Our results showed that a maternal low-protein diet promoted a decrease in AQP9 levels in LP animals at both PND7 and PND14. We recently reported that maternal protein restriction decreases AQP9 expression in the IS and caput epididymis regions in prepuberal rat offspring (PND44) [29]. Corroborating these findings, Arrighi et al. (2010) [52] showed a reduction in AQP9 expression in the epididymis of adult rats subjected to malnutrition during perinatal life, and both studies associated these results with a reduction in testosterone levels.

AQP9 expression is mostly modulated by dihydrotestosterone (DHT), the main metabolite of testosterone, which is abundantly present in the testicular fluid that arrives to the epididymis through efferent ducts [55]. However, before the first wave of testicular fluid, at PND15, the increase in AQP9 mRNA expression and protein levels may have different mechanisms of regulation [56]. Data from our research group showed that maternal protein restriction reduced testosterone conversion to DHT by 5α-reductase actions on these same postnatal days (7 and 14) (unpublished data).

We suggest that the lower expression of AQP9 observed in LP animals on PND7 and PND14 could be related to the decrease in the MVD index since there was a reduction in the bloody supply that arrives to the epididymis as well as in testosterone. In addition, less testosterone would be converted into DHT due to the lower expression of 5α-reductase (unpublished data). At the beginning of epididymis postnatal development, this may lead to a permanent effect on fluid reabsorption, mainly in the initial segment and the caput epididymis; this could promote changes in the epithelial structure, thus compromising epididymal functionality in adulthood. The imbalance in AQP9 expression can change the water absorption mechanism and consequently alter the epididymal microenvironment [53,55], representing one of the factors that contribute to changes in sperm quality already found in adults subjected to this experimental model [16]. 

Although there are few studies on the subject, it is known that male rat offspring from mothers fed a low-protein diet during pregnancy had alteration on sperm quality (~50% has shown a decrease in fertility rate compared to controls) [57]. It is well established that the combined secretory and absorptive activities of the epididymal epithelial cells are responsible for the creation of the specialized luminal microenvironment that promotes fertility in the sperm population [58]. The presence of AQPs, channel proteins that facilitate rapid passive movement of water across cell membranes, is of major importance in the male genital ducts, as is the epididymis, where water movements mainly take place [59]. Although homozygous AQP9-/- knockout mice were demonstrated to be fertile with normal sperm motility and morphology [60], other studies have shown that alterations in the expression, function, and regulation of AQPs and its interaction with other proteins could be the basis of some types of male subfertility. In this sense, research has shown that AQP9 is responsible for H_2_O_2_ diffusion in the luminal microenvironment, and analysis of functional sperm parameters showed that H_2_O_2_ treatment reduces the vitality and progressive motility of sperm [61,62]. Another example of AQP interactions was shown in initial segment-specific Pten knockout mouse. PTEN is responsible for regulating a broad range of transporters in many organs and cell types responsible for the formation and regulation of the epididymal luminal microenvironment, as ion and water transporters including Na+-K+ ATPase subunits and AQP9. The loss of PTEN changed the transporter activity, resulting in alteration of the luminal fluid microenvironment that possibly led to male infertility [63].

A normal epididymal structure was observed in both groups at PND7 and PND14. However, there was a significant decrease in the mean values of the epididymal duct and luminal diameters, whereas the epithelium was thinner in LP animals, mainly in the IS, caput and corpus regions. An association between decreased plasma testosterone levels and changes in the diameter of the epididymis has been shown. Therefore, androgen deprivation over time can be related to a decreased MVD index and lower blood supply, possibly resulting in a reduction in the epididymal tubule diameter in all epididymal segments and a decrease in the height of epithelial cells in the IS and caput of adult rats [64].

Our data support the hypothesis that a low-protein diet during gestation and lactation alters VEGFa, VEGFr-2 and AQP9 expression, as well as the epididymal microvascular density, leading to impairment in the epididymal postnatal development of the offspring.

It is not still clear which are the mechanisms and molecule pathways essential for epididymis function that are affected by maternal protein restriction. Our study shows changes in the levels of some proteins involved in the epididymal intraluminal environment and suggests that this may be one of the factors responsible for the change in sperm quality previously described by other authors [16,57,65]. Thus, it seems interesting to repeat this experimental protocol, to investigate molecules involved in the epididymal luminal environment formation and maintenance during postnatal development, and proteins secreted into the epididymal luminal environment, in addition to the molecules known to create the epididymal luminal microenvironment, e.g., ATPase, apolipoprotein, Ros-1. Finally, the possibility of monitoring the possible effects on successive generations could add important and useful information to understand other topics of the fetal programming theories.

## 5. Conclusions

Maternal protein restriction changes epididymal morphogenesis during the onset of postnatal development. These changes are associated with an impaired bloody supply and alterations in the expression of proteins required for angiogenesis and for the correct formation and maintenance of the epididymal microenvironment. Thus, our data suggest that these alterations during the early stages of epididymal development may be related to changes in sperm quality, which were already reported in adult animals.

## Figures and Tables

**Table 1:** Composition of the food provided to the animals during the gestation and lactation periods.

Components *	Normoprotein (17% Protein)	Low-Protein (6% Protein)
Casein (84% of protein) **	202.00	71.50
Corn flour	397.00	480.00
Dextrin	130.50	159.00
Sucrose	100.00	121.00
Soy oil	70.00	70.00
Fiber (microcellulose)	50.00	50.00
Mineral blend ***	35.00	35.00
Vitamin blend ***	10.00	10.00
L–cystine	3.00	1.00
Choline chloride	2.50	2.50

* Diet for rodent during gestation- AIN-93G. ** Values corrected according to casein. *** Following AIN-93G.

**Table 2:** Epididymal morphometry at PND7.

Parameters (µm)	PND7							
	IS		Caput		Corpus		Cauda	
	NP	LP	NP	LP	NP	LP	NP	LP
Tubular diameter	23.87 ± 0.37	21.86 ± 0.43 *	25.90 ± 0.57	23.33 ± 0.27 *	27.97 ± 0.25	25.58 ± 0.82 *	35.85 ± 1.45	32.33 ± 2.53
Epithelial height	7.73 ± 0.22	7.22 ± 0.06	7.15 ± 0.29	6.78 ± 0.18	7.78 ± 0.01	7.268 ± 0.07	8.579 ± 0.83	7.14 ± 0.43
Luminal diameter	7.49 ± 0.15	6.70 ± 0.18 *	10.97 ± 0.51	9.29 ± 0.68	12.10 ± 0.34	10.60 ± 1.27	19.23 ± 0.45	18.01 ± 3.11

NP N = 4 and LP N = 4; Data expressed as the mean ± S.E.M. * *p* < 0.05. *T*-test.

**Table 3:** Epididymal morphometry at PND14.

Parameters (µm)	PND14							
	IS				IS			
	NP	LP	NP	LP	NP	LP	NP	LP
Tubular diameter	28.47 ± 0.80	25.80 ± 1.30	33.55 ± 2.24	29.41 ± 1.26	40.18 ± 1.05	32.46 ± 1.04 *	39.82 ± 2.17	38.29 ± 1.97
Epithelial height	9.50 ± 0.28	8.17 ± 0.39 *	8.90 ± 0.99	7.47 ± 0.60	11.85 ± 0.28	9.17 ± 0.67 *	10.93 ± 1.72	8.07 ± 0.23
Luminal diameter	9.22 ± 0.62	8.56 ± 0.51	14.16 ± 1.51	14.18 ± 0.81	15.06 ± 0.62	14.52 ± 0.73	19.02 ± 1.86	21.99 ± 2.32

NP N = 4 and LP N = 4; Data expressed as the mean ± S.E.M. * *p* < 0.05. *T*-test.

## Supplementary Materials

The following are available online at https://www.mdpi.com/2073-4409/8/9/1094/s1, Figure S1: Epididymis sections of the initial segment, caput, corpus and cauda from the NP and LP animals at PND7, stained with hematoxylin-eosin., Table S1: Epididymis sections of the initial segment, caput, corpus and cauda from the NP and LP animals at PND14, stained with hematoxylin-eosin.
