# Supplementary material for: Maternal Protein Restriction Modulates Angiogenesis and AQP9 Expression Leading to a Delay in Postnatal Epididymal Development in Rat
Source: Cells. 2019 Sep 17;8(9):1094. doi: 10.3390/cells8091094 (PMC6770568; doi:10.3390/cells8091094)
Supplement: Supplementary file 1 [file cells-08-01094-s001.pdf]

Supplementary

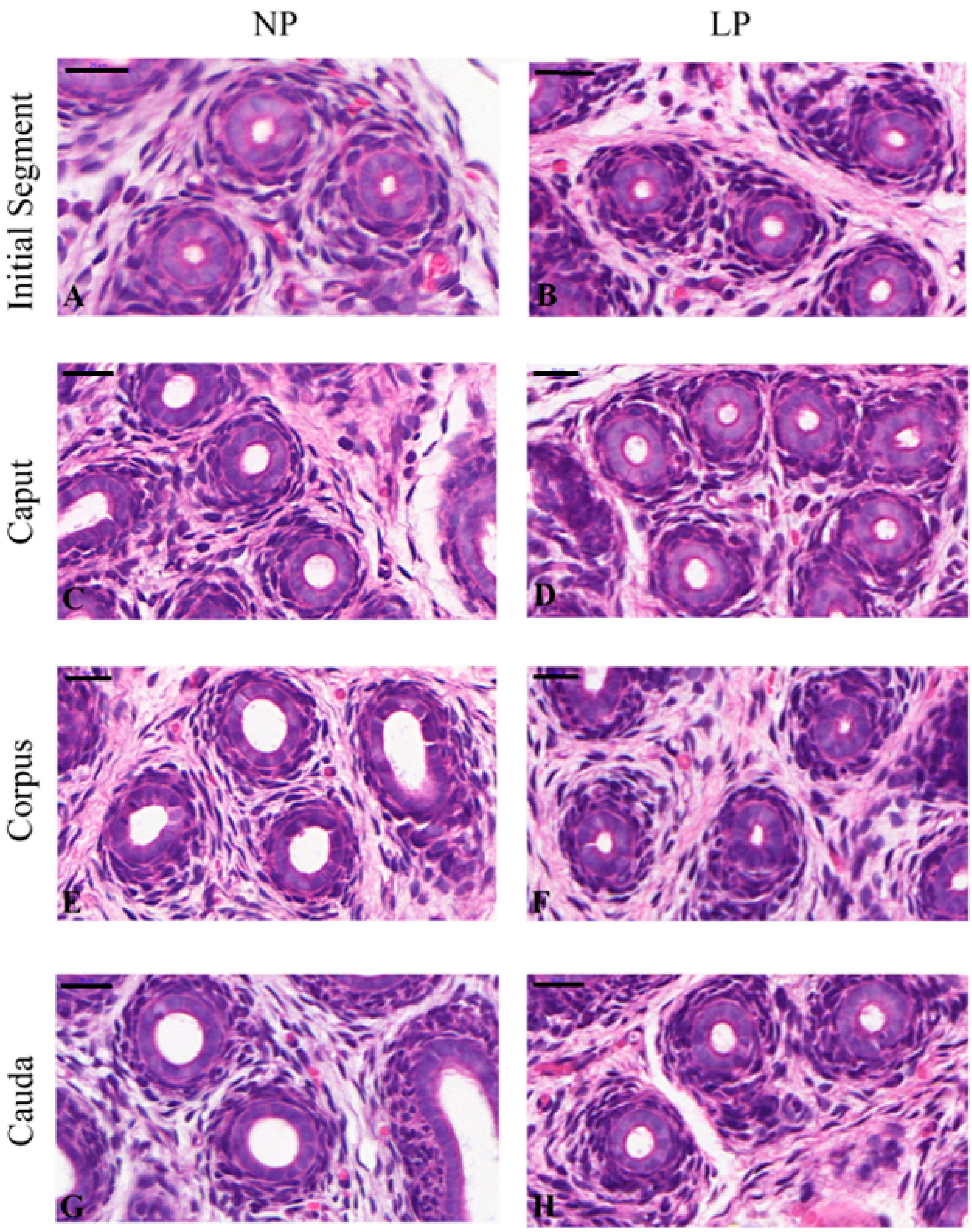

Figure S1. Epididymis sections of the initial segment, caput, corpus and cauda from the NP and LP animals at PND7, stained with hematoxylin-eosin. NP = normoprotein animals; LP = low-protein animals. Scale bar = 20 μm.

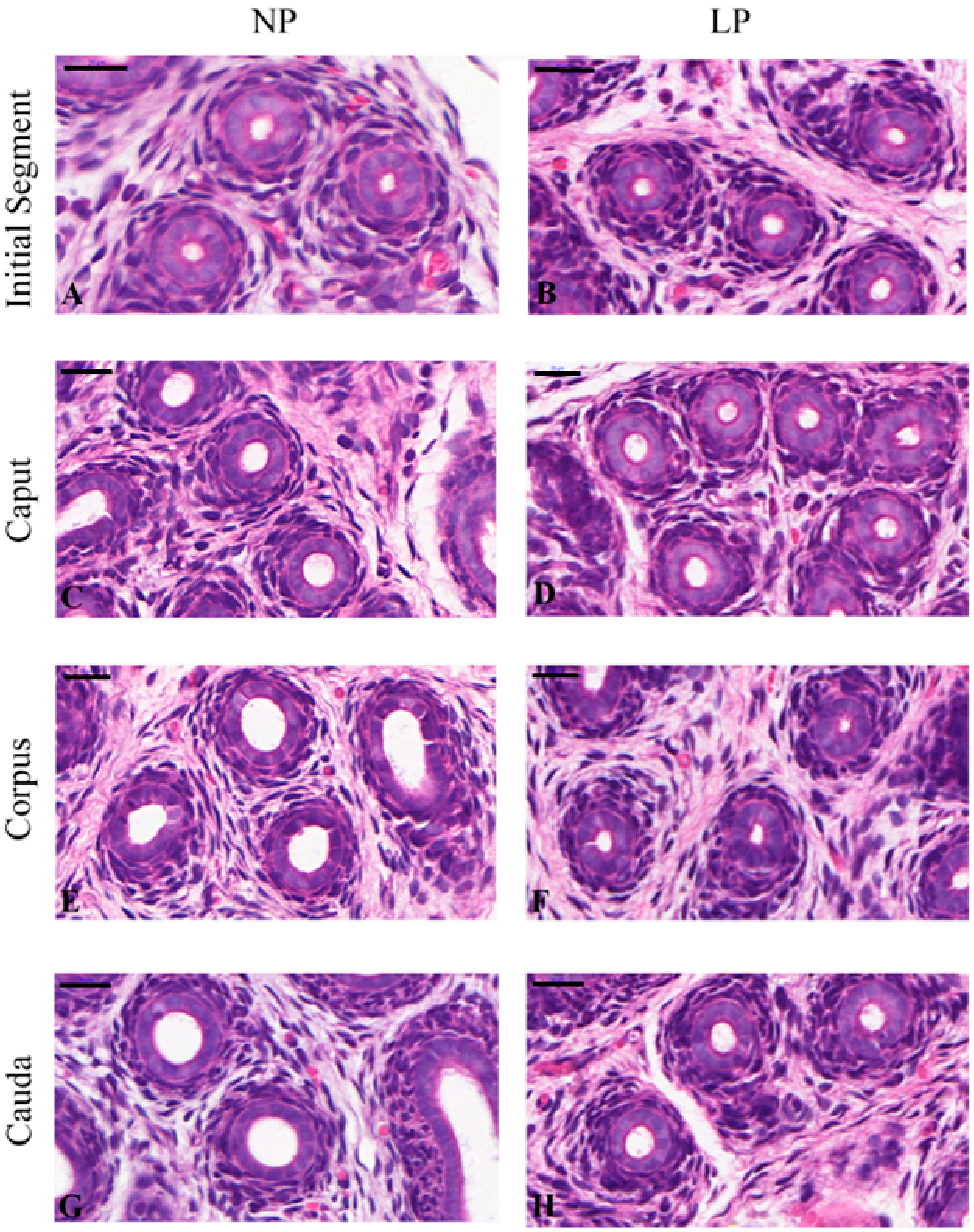

Figure S2. Epididymis sections of the initial segment, caput, corpus and cauda from the NP and LP animals at PND14, stained with hematoxylin-easin. NP = nor moprotein animals; LP = low-protein animals. Scale bar = 20 μm.
